# A deep transfer learning framework for the automated assessment of corneal inflammation on in vivo confocal microscopy images

**DOI:** 10.1371/journal.pone.0252653

**Published:** 2021-06-03

**Authors:** Fan Xu, Yikun Qin, Wenjing He, Guangyi Huang, Jian Lv, Xinxin Xie, Chunli Diao, Fen Tang, Li Jiang, Rushi Lan, Xiaohui Cheng, Xiaolin Xiao, Siming Zeng, Qi Chen, Ling Cui, Min Li, Ningning Tang

**Affiliations:** 1 Guangxi Health Commission Key Laboratory of Ophthalmology and Related Systemic Diseases Artificial Intelligence Screening Technology, Ophthalmology Department, the People’s Hospital of Guangxi Zhuang Autonomous Region, Nanning, Guangxi, China; 2 China-ASEAN Information Harbor, Nanning, Guangxi, China; 3 Guangxi Colleges and Universities Key Laboratory of Intelligent Processing of Computer Image and Graphics, Guilin University of Electronic Technology, Guilin, Guangxi, China; 4 Guangxi Key Laboratory of Embedded Technology and Intelligent Systems, Guilin University of Technology, Guilin, Guangxi, China; 5 School of Computer Science and Engineering, South China University of Technology, Guangzhou, Guangdong, China; University of Craiova, ROMANIA

## Abstract

**Purpose:**

Infiltration of activated dendritic cells and inflammatory cells in cornea represents an important marker for defining corneal inflammation. Deep transfer learning has presented a promising potential and is gaining more importance in computer assisted diagnosis. This study aimed to develop deep transfer learning models for automatic detection of activated dendritic cells and inflammatory cells using in vivo confocal microscopy images.

**Methods:**

A total of 3453 images was used to train the models. External validation was performed on an independent test set of 558 images. A ground-truth label was assigned to each image by a panel of cornea specialists. We constructed a deep transfer learning network that consisted of a pre-trained network and an adaptation layer. In this work, five pre-trained networks were considered, namely VGG-16, ResNet-101, Inception V3, Xception, and Inception-ResNet V2. The performance of each transfer network was evaluated by calculating the area under the curve (AUC) of receiver operating characteristic, accuracy, sensitivity, specificity, and G mean.

**Results:**

The best performance was achieved by Inception-ResNet V2 transfer model. In the validation set, the best transfer system achieved an AUC of 0.9646 (P<0.001) in identifying activated dendritic cells (accuracy, 0.9319; sensitivity, 0.8171; specificity, 0.9517; and G mean, 0.8872), and 0.9901 (P<0.001) in identifying inflammatory cells (accuracy, 0.9767; sensitivity, 0.9174; specificity, 0.9931; and G mean, 0.9545).

**Conclusions:**

The deep transfer learning models provide a completely automated analysis of corneal inflammatory cellular components with high accuracy. The implementation of such models would greatly benefit the management of corneal diseases and reduce workloads for ophthalmologists.

## Introduction

Inflammation and immune activation are the underlying process of a wide range of corneal diseases such as infective keratitis, immune and autoimmune corneal diseases [1,2]. Persistent inflammation can result in corneal opacity, significant visual impairment and even blindness. Patients with corneal inflammatory diseases may benefit from rational anti-inflammatory strategies. It is of high importance that the anti-inflammatory regimen should be dynamically adjusted according to the level of inflammatory response. Therefore, close monitoring of corneal inflammatory activity is warranted. However, symptoms and slit-lamp examination provide only rather rough estimates of ocular responses, making it difficult to accurately assess the inflammatory reaction and the effect of anti-inflammatory treatments.

In vivo confocal microscopy (IVCM) enables noninvasive analysis of different corneal layers in exquisite detail and allows in vivo detection of even subtle microstructural changes in pathological states [3,4]. IVCM image analysis reveals dendritic cells (DCs) activation and inflammatory cells infiltration in pathologic and infectious conditions such as dry eye [5,6], infectious keratitis of various aetiologies [7,8], and contact lens-induced corneal changes [9]. The inflammatory cellular components are considered as excellent indicators of inflammatory activity and clinical severity [5,8]. The activated DC, a type of antigen-presenting cell that initiates proinflammatory reactions in the cornea, is associated with the severity of dry eye disease [5], neuro-inflammatory disease [10] and corneal ulcer [11]. The round inflammatory cell, confirmed as the neutrophil in cornea [12], is associated with clinical outcomes of keratitis because of its ability to release cytokines that intensify inflammatory process [13]. Monitoring of these alterations contributes to optimize the tailored management of corneal inflammatory diseases. Manual analysis of the IVCM images, however, is extremely labor-intensive, time consuming, requires expertise, and is inherently subjective. Automation is therefore urgently needed and will facilitate standardized analyses among different centers.

Recently, artificial intelligence (AI) approaches such as deep learning [14] have demonstrated extraordinary performance in computer vision and medical image analysis tasks. However, a deep learning model requires training with millions of data points before making a reliable classification. Deep transfer learning (DTL) is an approach in deep learning where knowledge is transferred from one model to another [15]. A transfer model is constructed using a pre-trained deep learning network as a fixed feature extractor for the task of interest. Transfer learning technique achieves an optimization that allows improved performance of classification models with a relatively small amount of data. This technique provides a new insight into solving the task of IVCM images automatic classification.

Several deep learning models have been applied to trace the nerve fiber and fungal hyphae in IVCM with impressive accuracy [16–19]. To the best of our knowledge, however, there have been no studies performed involving the automatic evaluation of corneal inflammation using IVCM images. The present study was designed to construct an effective diagnostic model using DTL approach, investigate five transfer network architectures, and compare their performance for detecting activated DCs and inflammatory cells using IVCM images.

## Materials and methods

### Data collection

A total of 4011 IVCM images of 48 eyes (35 eyes with keratitis, 7 eyes with dry eyes, and 6 eyes with pterygium) were included in our study. The data were collected from November 2018 to August 2020 at Guangxi Zhuang Autonomous Region People’s Hospital, China. We excluded poor-quality images such as those that were of low contrast, unfocused, or had other conditions that interfered with assessment. All images were anonymized prior to their use in the current investigation. This study was conducted in compliance with the Declaration of Helsinki and approved by the ethics committee of The People’s Hospital of Guangxi Zhuang Autonomous Region. Informed consent was waived because of the retrospective nature of the study and anonymized usage of images.

**Fig 1:**
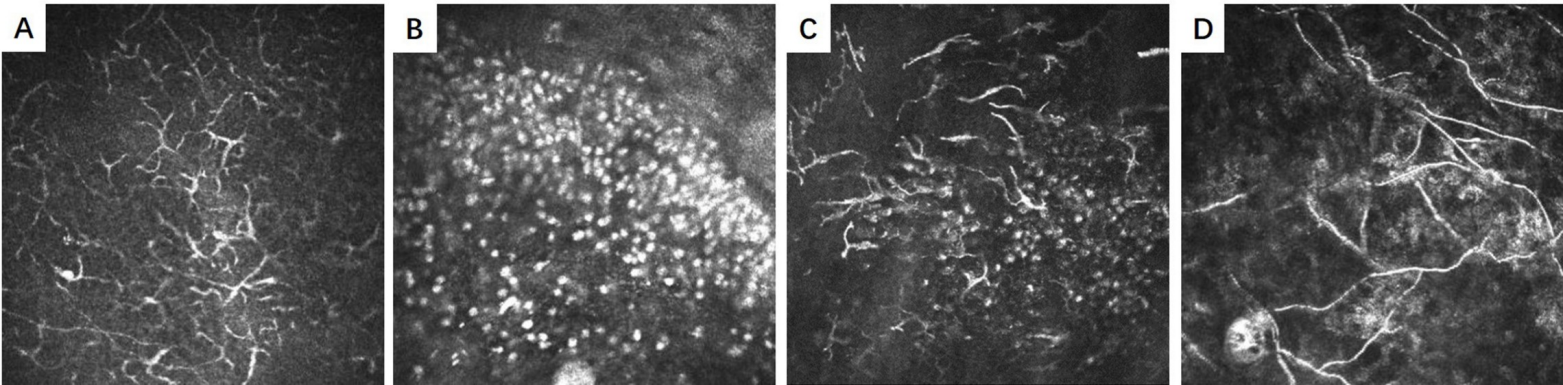
Activated dendritic cells, inflammatory cells and fungal hyphae shown in in vivo confocal microscopy images. (A) shows activated dendritic cells (DCs) that are characterized by hyperreflective branched structures, with long processes extending outwards in multiple directions from the cell body; (B) shows inflammatory cells that represent as small, round, bright hyperreflective cells; (C) shows the co-existence of activated DCs and inflammatory cells; (D) shows fungal hyphae that manifest as fine branched thread-like structures.

All images were taken using IVCM (HRT III/RCM Heidelberg Engineering, Germany). The data was initially assigned into training (collected from November 2018 to December 2019) and testing sets (collected in 2020). The testing set was employed for external validation. Each image was associated with two-level diagnostic labels for activated DCs (Positive: activated DCs; Negative: no activated DCs) and inflammatory cells (Positive: inflammatory cells; Negative: no inflammatory cells). Activated DCs were characterized by hyperreflective branched structures, with long processes extending outwards in multiple directions from the cell body (Fig 1A and 1C). Inflammatory cells were represented as small, round, bright hyperreflective cells (approximate 10 microns in diameter) and often accumulated at the lesion site (Fig 1B and 1C). In addition, 540 IVCM images containing fungal hyphae (Fig 1D) were included as negative samples.

### Labelling and preprocessing

The anonymous IVCM images were independently analyzed by three specialists with over 10 years of experience in cornea examinations. A ground-truth label was assigned to each image when consistent diagnostic outcomes were achieved by the three ophthalmologists. Any level of disagreement was adjudicated by another cornea specialist with 20 years of experience.

The pixel values of the images were normalized into range [0, 1] before being input to the models. The original IVCM images were resized to a standard resolution of 224 × 224 pixels to match the input size of the networks. Data augmentation, a technique commonly used to increase the diversity of data, was performed in the study. Specifically, flipping and 90° rotation were applied to the images in the training set to increase the amount of training data by fourfold.

### Deep transfer learning model and training

**Fig 2:**
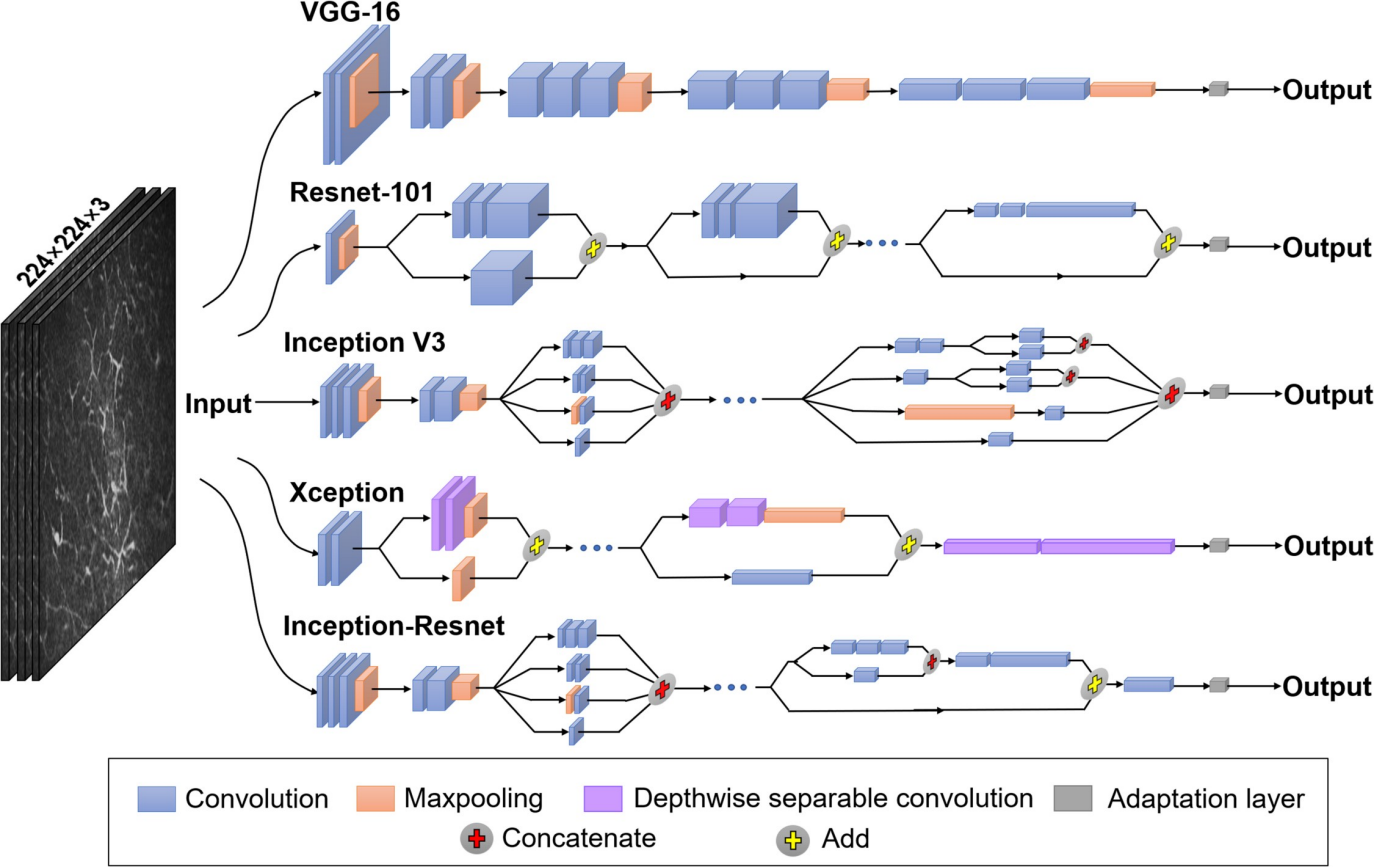
An overview of the five pre-trained networks architectures used in this study. The networks take images of 224 × 224 pixels as input and process the images through layers of nonlinear operations such as convolution and pooling. The arrows indicate the direction of flow. All convolution layers of the VGG-16 networks are depicted, but the intermediate repetitive layers of the other networks are omitted for simplicity. The final fully-connected layer was removed, replaced by an adaption layer.

**Fig 3:**
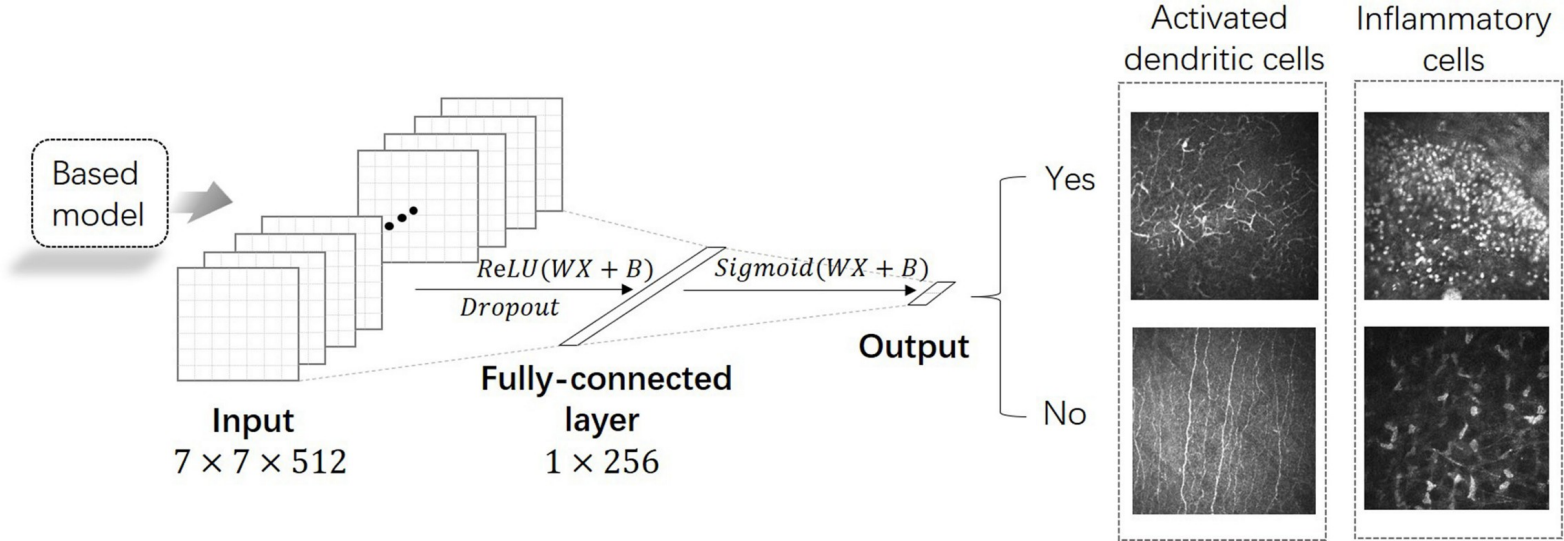
The adaptation layer of the deep transfer learning model. The front part of the pre-trained networks was adopted as the fixed feature extractor for our training dataset. We added an adaptation layer formed by two fully-connected layers that used the output vector of the fixed feature extractor as input. The activation function of the first fully-connected layer is ReLU, and the fully-connected output is a sigmoid function.

In this study, network-based DTL was performed to overcome the deficit of training data for deep learning. Network-based DTL refers to the reuse the partial network structure and parameters that pre-trained in the source task, transfer it to be a part of deep neural network which used in the target task [20]. For the source task, we use five network architectures, namely: Visual Geometry Group-16 (VGG-16), Residual Network-101 (ResNet-101), Inception V3, Xception, and Inception-ResNet V2 (Fig 2). In each network, we used the front-layers and connection parameters pre-trained on the ImageNet dataset (a large dataset contains 1.2 million images with 1000 categories) [21]. The final fully-connected layers were removed, and the front part of the networks was adopted as the fixed feature extractor for our training dataset. Then we added an adaptation layer formed by two fully-connected layers FC_α_ and FC_β_ that used the output vector *Y*_*fixed*_ of the fixed feature extractor as input (Fig 3). Note that *Y*_*fixed*_ was obtained as a complex non-linear function of potentially all input pixels and captured intermediate image representation. The computation proceeded as follows:

$$Y_{\alpha }=\sigma (W_{\alpha }Y_{fixed}+B_{\alpha })$$ (1)

σ(x)=max(0,x) (2)

$$\tilde{Y_{\alpha }}=r.Y_{\alpha }$$ (3)

r∼Bernoulli(p) (4)

$$Y_{\beta }=\psi (W_{\beta }\tilde{Y_{\alpha }}+B_{\beta })$$ (5)

$$\psi (x)=1/(1+e^{-x})$$ (6)

Formulas (1) and (2) are the computing process of FC_α_, where *W*_*α*_ denotes the trainable weight of FC_α_, *B*_*α*_ denotes the trainable bias of FC_α_, and *σ*(*x*) is the “ReLU” activation function. Formulas (3) and (4) denote dropout of FC_α_, where “*∙*” denotes an element-wise product, *Bernoulli*(*p*) is the “Bernoulli” function, p is the dropout rate, and r is a vector of independent Bernoulli random variables that has probability p of being 1. This vector is multiplied in an element-wise manner with *Y*_*α*_ to create the thinned outputs $\tilde{Y_{\alpha }}$. Formulas (5) and (6) are the computing process of FC_β_, where *W*_*β*_ denotes the weight of FC_β_, *B*_*β*_ denotes the bias of FC_β_, *ψ*(*x*) is the “Sigmoid” activation function, and *e* refers to the natural logarithm.

The weights of the networks were fine-tuned by continuing the backpropagation. During training, the binary cross entropy loss metric was optimized through stochastic gradient descent method. The initial value of learning rate was set to 0.001 and the learning rate was adjusted through self-adaptation mechanism [22]. The discounting factor for the history/coming gradient, the momentum and the centered Boolean were set as 0.9, 0.0 and False, respectively. A batch size of 32 was used for the training. The training epoch of VGG-16, ResNet-101, Inception V3, Xception, and Inception-ResNet V2 models for detecting activated DCs were 26, 27, 18, 31, and 18, respectively; and that for detecting inflammatory cells were 22, 26, 12, 16, and 10, respectively. All experiments were conducted on an NVIDIA Tesla T4 Tensor Core GPU. All models were implemented using Keras, Tensorflow-2.3-gpu. Programs were written in the Python programming language (Python 3.7, Python Software Foundation). The detailed characteristics of the transfer models were recorded in S1 Table.

The classical architecture of VGG-16 network consists of 13 convolution layers, five pooling layers, and three fully-connected layers [23]. In convolutional layers, 3 × 3 kernel-sized filters are used to convolve the input images and generate hierarchical feature maps. Max-pooling strategy with a dropout rate at 0.5 is adopted on the pooling layers (also called down-sampling layers) to reduce the dimensionality of the feature.

The ResNet-101 presents a residual learning framework to ease the training of networks [24]. It consists of multiple residual blocks connected in series, with each block containing a shortcut route and a residual route [25]. Down-sampling is performed by convolution layers with a stride of two. In ResNet-101 architecture, the size of feature map is reduced by half, while the number of feature map doubles, thereby maintaining the network complexity.

The characteristic of Inception V3 lies in extracting multiple features in the same layer [26]. The input is transferred to various extraction methods such as convolution kernels of different sizes (for example, 1×1, 1×3, 1×7 et al). Concatenating operation is used to integrate the features.

Xception is proposed as an improvement to the Inception V3. In Xception networks, depthwise separable convolution is substituted for the general convolution [27], leading to higher performance at relatively low computational cost.

Inception-ResNet V2 is a modified version of the Inception model, which incorporates the idea of residual learning [28]. In each block, 1 × 1 convolution is added before addition operation, playing the role of filter-expansion layer to scale up the dimensionality of the filter bank. The network has the advantages of both Inception and ResNet and thereby improving the training efficiency.

### Comparison with human ophthalmologists

To evaluate the performance of our transfer networks, we recruited two human ophthalmologists to read the images independently. Ophthalmologist A had three years of experience and the ophthalmologist B had one years of experience in IVCM image analysis. Both ophthalmologists were blinded to the ground-truth. The results of ophthalmologists were compared with those of the transfer models in detecting activated DCs and inflammatory cells in the external testing sets.

### Experimental setup

**Fig 4:**
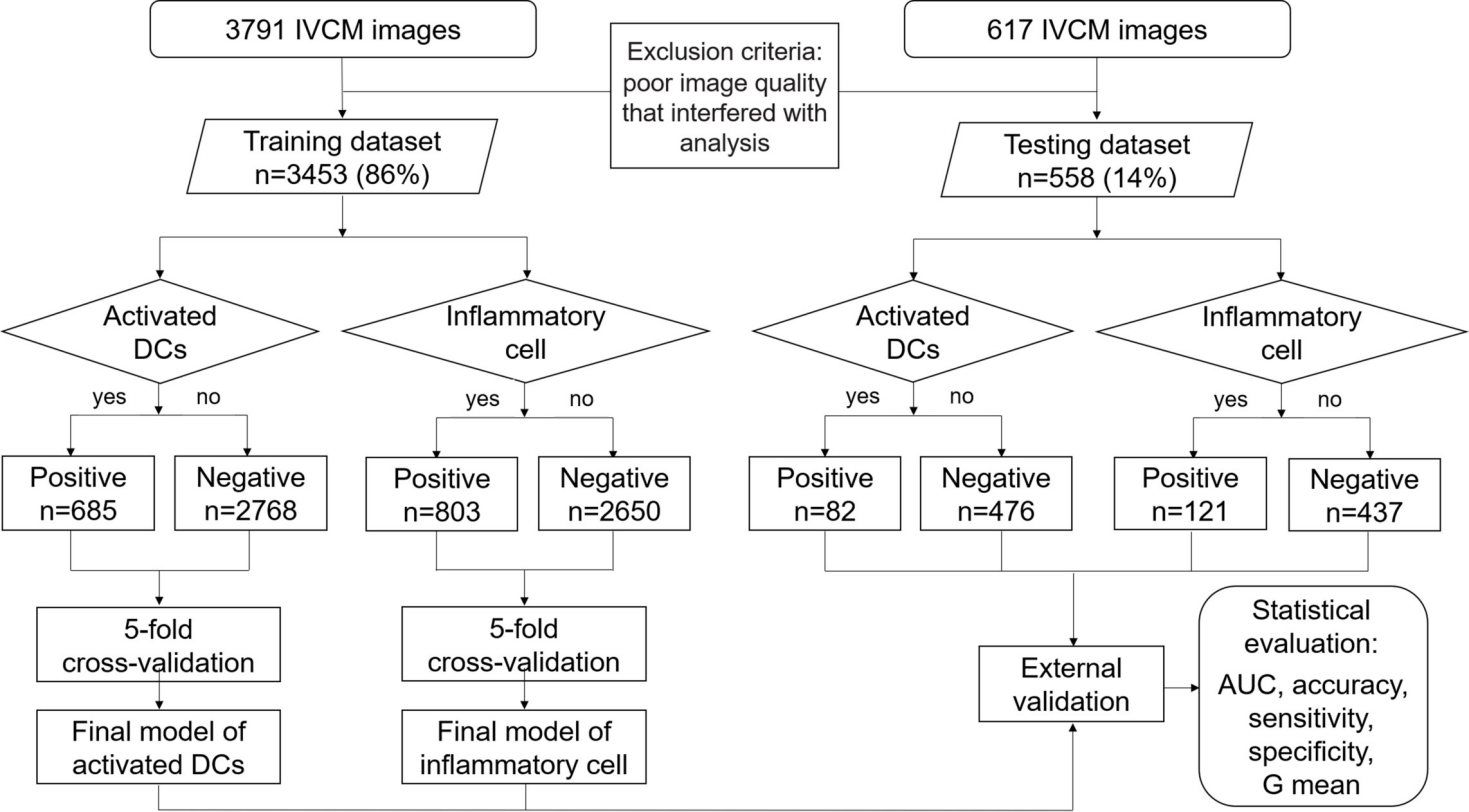
The detailed model building process. The training dataset consisted of 3453 (86%) in vivo confocal microscopy (IVCM) images. Five-fold cross-validation was used for parameters tuning. The testing dataset comprised 558 (14%) IVCM images and was used to perform external validation. Area under the curve (AUC), accuracy, sensitivity, specificity, and G mean were calculated to evaluate the final performance of the deep transfer learning models.

Five-fold cross-validation was used to select the tuning parameters. With this approach, training data were randomly split into five subsets. Each time, four subsets were used as training set and one was withheld as validation set. The process was iterated five times until each of the five subsets was used as a validation dataset once [29]. The final models were trained on the entirety of training dataset, and were used to perform external validation on external testing set (Fig 4).

The classification outcomes were represented as confusion matrices. Accuracy, sensitivity, specificity, and G mean of the five DTL models and two ophthalmologists were calculated as follows:

$$Accuracy=\frac{TP+TN}{TP+FP+TN+FN}$$

$$Sensitivity=\frac{TP}{TP+FN}$$

$$Specificity=\frac{TN}{TN+FP}$$

$$G\;mean=\sqrt{Sensitivity\times Specificity}$$

where TP, FP, TN, and FN represented the number of true positive, false positive, true negative, and false negative samples, respectively.

A receiver operating characteristic (ROC) curve that plots the true positive rate (i.e., sensitivity) against the false positive rate (i.e., 1-specificity) was generated to evaluate every model and ophthalmologist on external validation sets. The closer the ROC curve was to the upper left border, the higher the overall accuracy of the test. The area under the curve (AUC) was calculated for each ROC curve.

### Statistical analysis

Data were analyzed using SPSS (SPSS Version 11.0, IBM-SPSS Inc., Chicago, IL, USA) and MedCalc (MedCalc version 19.7.2, MedCalc Inc., Ostend, Belgium). The AUC represents the overall performance of the algorithm, where one could distinguish between non-informative (AUC = 0.5), less accurate (0.5 < AUC ≤ 0.7), moderately accurate (0.7 < AUC ≤ 0.9), highly accurate (0.9 < AUC < 1) and perfect discrimination (AUC = 1). The AUC was compared with the chance level (AUC = 0.5). The statistical significance P < 0.05 was considered statistically significant. Pair-wise comparisons of ROC between the models were made by MedCalc software according to the method proposed by Delong DM et al [30]. The accuracy, sensitivity and specificity of models and ophthalmologists were compared by chi-square test. The Bonferroni test was used to correct for multiple comparisons. The significance was set at 0.05/N, where N is the number of tests used.

## Results

**Fig 5:**
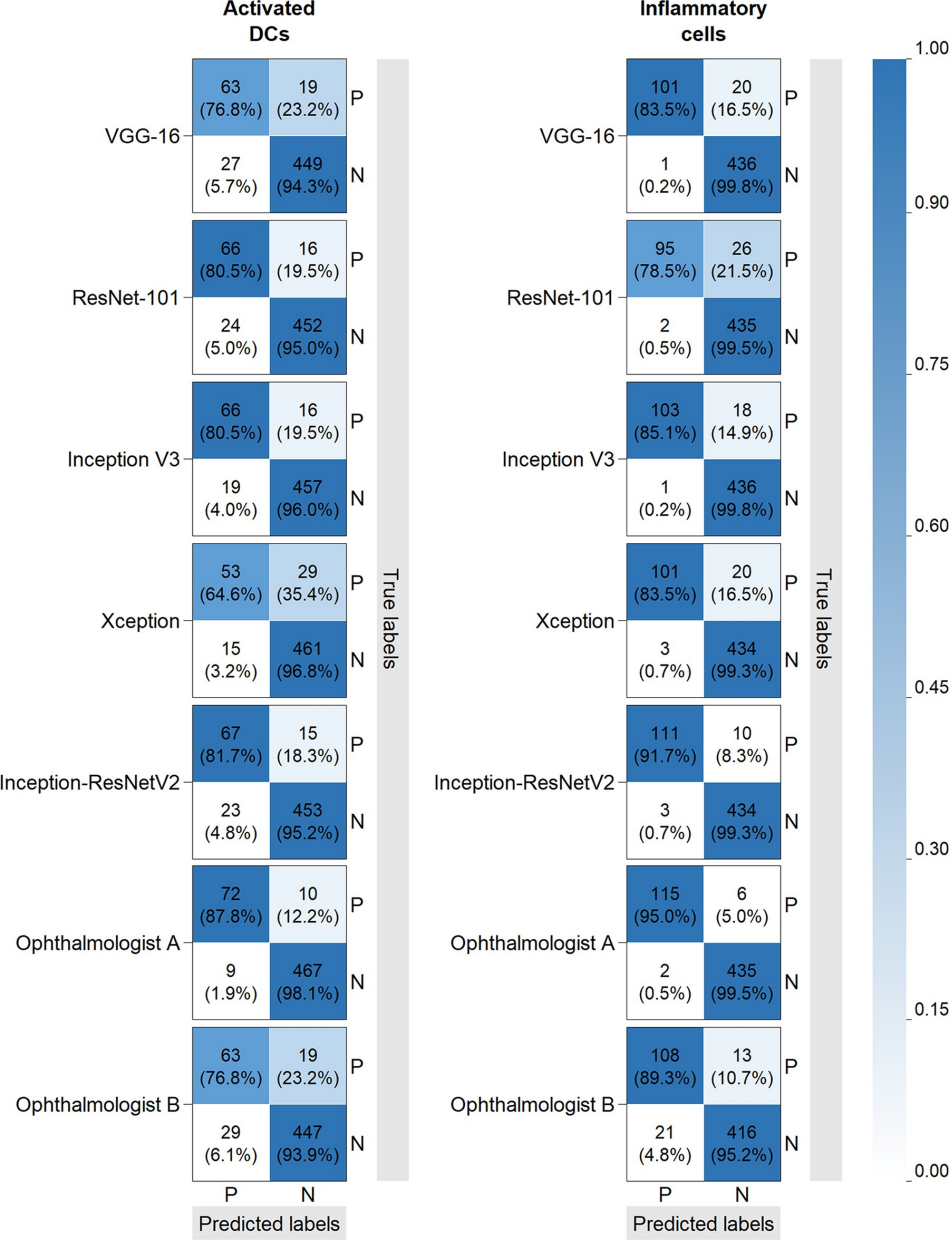
Confusion matrices of external validation in detecting activated dendritic cells (DCs) and inflammatory cells. DCs, dendritic cells; P: Positive; N: Negative. Confusion matrices of deep transfer learning models and ophthalmologists in detecting activated dendritic cells (DCs) and inflammatory cells are shown. True positive, true negative, false positive and false negative rates are calculated. Matrix cells are colored according to the rates.

A total of 4011 IVCM images were used to train and test the performance of the DTL models, after the exclusion of 397 images due to poor quality that interfered with reliable interpretation. Of the 4011 images, 3453 constituted the training set and 558 made up the external testing set (Fig 4). External validation results of five models and two ophthalmologists are shown in the confusion matrices (Fig 5). The performance of classifiers in internal validation study were likely to be optimistic (shown in S2 Table), and better than their performance when applied to external data; therefore, results of external validation were considered as the primary evaluation indicators to assess the applicability of the models.

### Detection of activated dendritic cells

**Fig 6:**
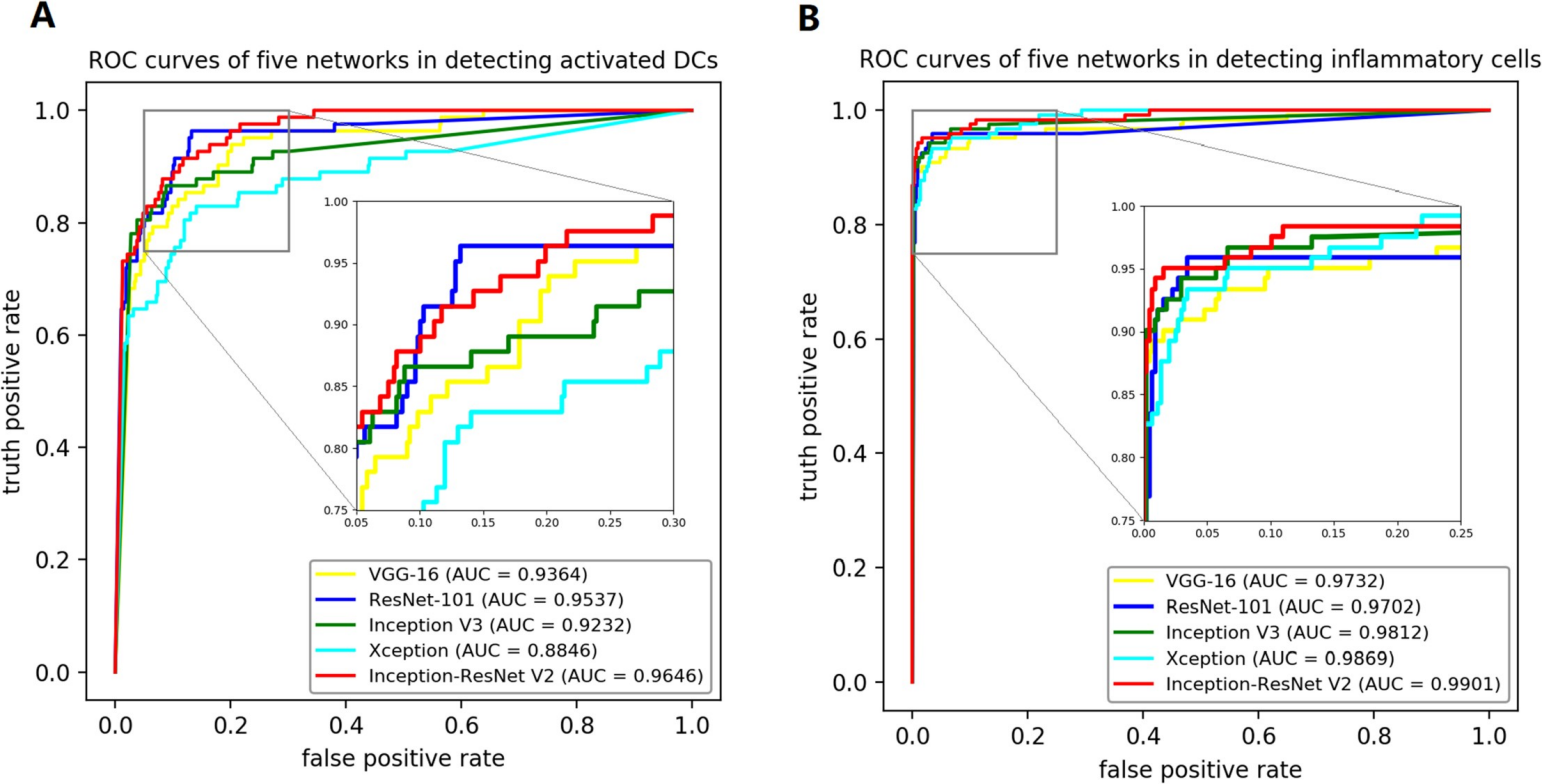
Performance of the deep transfer learning (DTL) models in external validation datasets. (A) shows the receiver operating characteristic (ROC) curves of five transfer models in identifying activated DCs. The area under the curve (AUC) ranged from 0.8846 to 0.9646. (B) shows the ROC curves of five transfer models in identifying inflammatory cells. The AUC ranged from 0.9701 to 0.9901.

The AUC of DTL models in detecting activated DCs ranged from 0.8846 to 0.9646 in the external validation (Table 1). ROC curves showed that Inception-ResNet V2 transfer model and ResNet-101 transfer model exhibited excellent diagnostic efficiency (Fig 6A). In this regard, Inception-ResNet V2 transfer network achieved an AUC of 0.9936 (accuracy, 0.9792; sensitivity, 0.9077; and specificity, 1) and 0.9646 (accuracy, 0.9319; sensitivity, 0.8171; specificity, 0.9517; and G mean, 0.8872) in training and testing datasets, respectively. Likewise, ResNet-101 transfer network detected activated DCs with an AUC of 0.9929 (accuracy, 0.9757; sensitivity, 0.9385; and specificity, 0.9865) and 0.9537 (accuracy, 0.9283; sensitivity, 0.8049; specificity, 0.9496; and G mean, 0.8743) in training and testing datasets, respectively.

**Table 1:** External validation results of five transfer networks and two ophthalmologists in detecting activated dendritic cells (DCs).

Transfer network	AUC			Accuracy	Sensitivity	Specificity	G mean
	mean	95% CI	AUC p				
VGG-16	0.9364	0.8993–0.9736	< 0.001	0.9176	0.7683	0.9433	0.8513
ResNet-101	0.9537	0.9216–0.9858	< 0.001	0.9283	0.8049	0.9496	0.8743
Inception V3	0.9232	0.8828–0.9637	< 0.001	0.9373	0.8049	0.9601	0.8791
Xception	0.8846	0.8364–0.9330	< 0.001	0.9211	0.6463	0.9685	0.7912
Inception-ResNet V2	0.9646	0.9365–0.9929	< 0.001	0.9319	0.8271	0.9517	0.8872
Ophthalmologist A	-	-	-	0.9659	0.8780	0.9811	0.9281
Ophthalmologist B	-	-	-	0.9140	0.7683	0.9391	0.8494

DCs, dendritic cells; AUC, area under the curve; CI, confidence interval; AUC p, P value of AUC.

Ophthalmologist A identified activated DCs with an accuracy of 0.9659 (sensitivity, 0.8780; specificity, 0.9811; and G mean, 0.9281) in testing dataset. Whereas ophthalmologist B had a diagnostic accuracy of 0.9140 (sensitivity, 0.7683; specificity, 0.9391; and G mean, 0.8494).

### Detection of inflammatory cells

The AUC of DTL models in detecting inflammatory cells ranged from 0.9701 to 0.9901 in the external validation (Table 2). The ROC curves of the five models were almost overlapping with each other (Fig 6B). Inception-ResNet V2 transfer model obtained the best performance, followed by Xception transfer model, as a close second. Inception-ResNet V2 transfer network achieved an AUC of 0.9957 (accuracy, 0.9826; sensitivity, 0.9600; and specificity, 0.9906) and 0.9901 (accuracy, 0.9767; sensitivity, 0.9174; specificity, 0.9931; and G mean, 0.9545) in training and testing datasets, respectively. Xception transfer network diagnosed inflammatory cells with an AUC of 0.9999 (accuracy, 0.9931; sensitivity, 0.9733; and specificity, 1) and 0.9869 (accuracy, 0.9588; sensitivity, 0.8347;specificity, 0.9931; and G mean, 0.9105) in training and testing datasets, respectively.

**Table 2:** External validation results of five transfer networks and two ophthalmologists in detecting inflammatory cells.

Transfer network	AUC			Accuracy	Sensitivity	Specificity	G mean
	mean	95% CI	AUC p				
VGG-16	0.9732	0.9530–0.9936	< 0.001	0.9624	0.8347	0.9977	0.9126
ResNet-101	0.9702	0.9487–0.9916	< 0.001	0.9498	0.7851	0.9954	0.8840
Inception V3	0.9812	0.9641–0.9984	< 0.001	0.9659	0.8512	0.9977	0.9215
Xception	0.9869	0.9726–1.0010	< 0.001	0.9588	0.8347	0.9931	0.9105
Inception-ResNet V2	0.9901	0.9776–1.0030	< 0.001	0.9767	0.9174	0.9931	0.9545
Ophthalmologist A	-	-	-	0.9857	0.9504	0.9954	0.9726
Ophthalmologist B	-	-	-	0.9391	0.8926	0.9519	0.9218

AUC, area under the curve; CI, confidence interval; AUC p, P value of AUC.

Both ophthalmologists achieved excellent discrimination of inflammatory cells. The accuracy of Ophthalmologist A and B in identifying inflammatory cells was 0.9857 (sensitivity, 0.9504; specificity, 0.9954; and G mean, 0.9726) and 0.9391 (sensitivity, 0.8926; specificity, 0.9519; and G mean, 0.9218), respectively.

### Statistical results

The difference between the AUC and the chance level (AUC = 0.5) was statistically significant (all P<0.001) (Tables 1 and 2), indicating that the AUC was clearly above chance.

For the detection of activated DCs, pairwise comparisons of AUC showed significant differences between Inception and Inception-ResNet V2 (P = 0.0062), and between Xception V3 and Inception-ResNet V2 (P = 0.0009). Significant differences for accuracy, sensitivity and specificity were found among groups (p = 0.014, 0.023 and 0.021 respectively); Bonferroni analyses indicated that the accuracies of VGG-16 and Xception V3 were significantly lower than that of ophthalmologist A, the sensitivity of Xception V3 was significantly lower than that of ophthalmologist A, and the specificity of VGG-16 was significantly lower than that of ophthalmologist A, while no differences were found among Inception-ResNet V2, Inception, ResNet-101 and ophthalmologist A.

For the detection of inflammatory cells, pairwise comparisons of AUC showed significant difference between VGG-16 and Inception-ResNet V2 (P = 0. 0051). The accuracy, sensitivity and specificity among groups differed statistically (p = 0.001, 0.003 and < 0.001, respectively). The accuracy of Inception-ResNet V2 was not statistically different from that of ophthalmologist A but significantly higher than that of ophthalmologist B. The sensitivity of ResNet-101 was significantly lower than that of ophthalmologist A. The specificities of all transfer models were not statistically significantly different from that of ophthalmologist A but significantly higher than that of ophthalmologist B.

## Discussion

In this study, DTL models based on five deep neural networks were used to provide a comprehensive view of the role of AI in detecting corneal inflammatory components using IVCM images. The results attained indicated the high efficacy of our transfer systems in identifying both activated DCs and inflammatory cells, which was comparable to that of human ophthalmologists. Given the advantages of technical feasibility and noninvasive nature of the image acquisition, the intelligent systems have great potential to facilitate the objective assessment of corneal inflammatory response and the elaboration of individualized treatment plans.

For activated DCs detection, Inception-ResNet transfer network displayed the best classification accuracy with an AUC of 0.9646. The images of fungal hyphae were incorporated into our datasets to increase heterogeneity of the distractors. Hyphae debris were morphologically similar to atypical DCs, which added to the difficulty of the classification task. In the false-positive group, nine images were misclassified due to fragmented hyphae with short irregular branches. Increasing the number of these error-prone images in the training set could potentially improve the classification performance.

For inflammatory cells detection, all five networks achieved good results, with Inception-ResNet showing the best performance. Morphologically, the inflammatory cells present as small, bright hyper-reflective round dots, and the characteristics are clear and stable. Therefore, the discrimination task was relatively straightforward, and the overall results were satisfactory.

There are a few studies that have investigated DTL’s detection ability on IVCM images. Lv et al [18] developed a DL system to detect fungal hyphea in IVCM images and achieved an AUC of 0.9875 with an accuracy of 0.9626. Wei et al [19] established a DL-based model to trace sub-basal corneal nerve and achieved an AUC of 0.96. Our study on DL’s detection of activated DCs and inflammatory cells in IVCM images also showed similar AUC and accuracy outcomes as those in previous reports. The neural networks provide optimal structure to learn and detect local features of complex IVCM image data.

A highlight of this study was the automated assessment of inflammatory cellular elements in IVCM images. The accuracy of transfer model was comparable to that of experienced ophthalmologist and better than under-experienced ophthalmologist. It has been reported that activated DCs and inflammatory cells are important biomarkers for monitoring inflammatory activity and clinical severity of corneal diseases [7,8]. Our DTL models proposed automated solutions for the evaluation of corneal inflammatory cellular components, which may support under-experienced ophthalmologists in decision making regarding the management of corneal diseases. Most importantly, the present study laid the foundations for the future investigation on the fully automated IVCM images analysis system.

The present study contrasted five pre-trained deep learning algorithms, and the results revealed that Inception-ResNet V2 transfer network has an advantage over the others. On the one hand, the hybrid Inception module of Inception-ResNet allows for multiple convolution and pooling operations in parallel. Concatenating the results gives rise to better image representation. On the other, the use of residual learning solves the degradation problem, which facilitates the training of substantially deeper neural networks. Hence, Inception-ResNet V2 could obtain outstanding performance.

One of the most innovative aspect of this study was the use of DTL technique. Although 4011 IVCM images were included in this study, the size of the dataset was not yet sufficient to meet the enormous demands for data to train a complete deep learning model. Transfer learning brings two main advantages: it requires far less data to achieve equal or even better performance, and it drastically shortens the training time. We divided the pre-trained network into two parts, the former part was transferred to be the feature extractor of the transfer model and the last layer was modified. It was based on the widely recognized view that the features extracted by the front-layers of the network are versatile [20,31]. Compared with traditional deep learning models [32], our approach improved the performance with a higher training start, a faster convergence rate and a better solution accuracy.

External validations were performed in this study. Images from the same patient had high similarity. Thus, random partition of training and validation datasets could lead to a biased high accuracy. To avoid this bias, the images of our external datasets were all obtained from new patients to warrant a stringent validation.

There are some limitations that should be considered. First, our models had high specificity but relatively low sensitivity. As we know, the infiltration of inflammatory cells and activation of DCs are non-specific manifestations of corneal inflammatory diseases. In this light, sensitivity is important because a false-negative result represents potentially denying a patient necessary special care. It is desired to introduce better models with higher sensitivity to ensure a minimum false-negative rate. Second, although we have collected as many images as possible, the clinical situation is undoubtedly more complicated. Therefore, more diversified images with heterogeneous background should be used to train robust models in follow-up studies. Third, binary classification models were established to detect whether an IVCM image exhibits inflammatory components, but it cannot calculate the density of the inflammation-related cells. There are two potential solutions to this problem: a density-based multi-classification method and an image segmentation method. For option one, images should be annotated as multi-category labels such as “no”, “low-density”, “medium-density” and “high-density”. Multi-class classification models will be used for the stratified analysis of density. For option two, image segmentation algorithms will be used to segment the inflammation-related cells from the IVCM images and thereby allows for automated cell counting. These approaches will be included in our future research direction.

In conclusion, this study developed accurate DTL-based models for detecting activated DCs and inflammatory cells using IVCM images. These findings suggest that DTL is useful in the objective assessment of corneal inflammation in a time-efficient manner. The models can be used as an assistant tool for the clinical assessment of corneal diseases.

## Supporting information

S1 Table Characteristics of five transfer networks used in this study. For each transfer model, a pre-trained network without the last fully-connected layer was used as a based model, of which the parameters were frozen and non-trainable. The parameters of the added adaptation layer were trainable. The depth of transfer networks is the sum of all layers, including convolution, pooling, batch normalization, activation, padding, concatenate, add, and fully-connected layers. The original images were resized to a standard resolution and were input to the based model. For each transfer model, the output size of based model was equal to the input size of top model.
(DOCX)

S2 Table Five-fold cross-validation results of five transfer networks during training process*. * The results are expressed as the mean ± standard deviations (the optimal value). AUC, area under the curve; DCs, dendritic cells.
(DOCX)

## Abbreviations

IVCM: in vivo confocal microscopy
DCs: dendritic cells
AI: Artificial intelligence
DTL: Deep transfer learning
VGG: Visual Geometry Group
ResNet: Residual Network
AUC: area under the curve
ROC: receiver operating characteristic
TP: true positive
FP: false positive
TN: true negative
FN: false negative
