# Supplementary material for: A deep transfer learning framework for the automated assessment of corneal inflammation on in vivo confocal microscopy images
Source: PLoS One. 2021 Jun 3;16(6):e0252653. doi: 10.1371/journal.pone.0252653 (PMC8174724; doi:10.1371/journal.pone.0252653)
Supplement: S1 Table — For each transfer model, a pre-trained network without the last fully-connected layer was used as a based model, of which the parameters were frozen and non-trainable. The parameters of the added adaptation layer were trainable. The depth of transfer networks is the sum of all layers, including convolution, pooling, batch normalization, activation, padding, concatenate, add, and fully-connected layers. The original images were resized to a standard resolution and were input to the based model. For each transfer model, the output size of based model was equal to the input size of top model. (DOCX) [file pone.0252653.s001.docx]

**S1 Table.** **Characteristics of five transfer networks used in this study.**

| Transfer network | Total parameters (×10^6^) | Trainable parameters (×10^6^) | Non-trainable parameters (×10^6^) | Depth | Input size of based model | Output Size of based model |
| --- | --- | --- | --- | --- | --- | --- |
| VGG-16 | 17.9 | 3.2 | 14.7 | 21 | 224-by-224 | 7-by-7 |
| ResNet-101 | 68.3 | 25.7 | 42.6 | 380 | 224-by-224 | 7-by-7 |
| Inception V3 | 34.9 | 13.1 | 21.8 | 314 | 224-by-224 | 5-by-5 |
| Xception | 46.5 | 25.7 | 20.8 | 135 | 224-by-224 | 7-by-7 |
| Inception-ResNet V2 | 64.1 | 9.8 | 54.3 | 783 | 224-by-224 | 5-by-5 |

For each transfer model, a pre-trained network without the last fully-connected layer was used as a based model, of which the parameters were frozen and non-trainable. The parameters of the added adaptation layer were trainable. The depth of transfer networks is the sum of all layers, including convolution, pooling, batch normalization, activation, padding, concatenate, add, and fully-connected layers. The original images were resized to a standard resolution and were input to the based model. For each transfer model, the output size of based model was equal to the input size of top model.
