# Supplementary material for: A deep transfer learning framework for the automated assessment of corneal inflammation on in vivo confocal microscopy images
Source: PLoS One. 2021 Jun 3;16(6):e0252653. doi: 10.1371/journal.pone.0252653 (PMC8174724; doi:10.1371/journal.pone.0252653)
Supplement: S2 Table — * The results are expressed as the mean ± standard deviations (the optimal value). AUC, area under the curve; DCs, dendritic cells. (DOCX) [file pone.0252653.s002.docx]

**S2 Table. Five-fold cross-validation results of five transfer networks during training process*.**

| Transfer network | AUC | Accuracy | Sensitivity | Specificity |  |
| --- | --- | --- | --- | --- | --- |
| Activated DCs | | | | | |
| VGG-16 | 0.9618 ± 0.0163 (0.9671) | 0.9502 ± 0.0324 (0.9618) | 0.8538 ± 0.0737 (0.8923) | 0.9796 ± 0.0032 (0.9821) |  |
| ResNet-101 | 0.9903 ± 0.0032 (0.9929) | 0.9692 ± 0.0084 (0.9757) | 0.9061 ± 0.0504 (0.9385) | 0.9814 ± 0.0022 (0.9865) |  |
| Inception V3 | 0.9796 ± 0.0096 (0.9821) | 0.9452 ± 0.0163 (0.9583) | 0.8649 ± 0.0607 (0.8923) | 0.9716 ± 0.0019 (0.9776) |  |
| Xception | 0.9782 ± 0.0112 (0.9825) | 0.9649 ± 0.0132 (0.9722) | 0.8695 ± 0.0548 (0.8923) | 0.9918 ± 0.0026 (0.9955) |  |
| Inception-ResNet V2 | 0.9914 ± 0.0052 (0.9936) | 0.9711 ± 0.0109 (0.9792) | 0.8737 ± 0.0527 (0.9077) | 0.9975 ± 0.0013 (1.0000) |  |
| Inflammatory cells | | | | | |
| VGG-16 | 0.9866 ± 0.0132 (0.9949) | 0.9717 ± 0.0228 (0.9792) | 0.9235 ± 0.0609 (0.9467) | 0.9856 ± 0.0069 (0.9906) |  |
| ResNet-101 | 0.9905 ± 0.0068 (0.9932) | 0.9838 ± 0.0107 (0.9861) | 0.9384 ± 0.0401 (0.9467) | 0.9928 ± 0.0093 (1.0000) |  |
| Inception V3 | 0.9936 ± 0.0051 (0.9988) | 0.9875 ± 0.0197 (0.9931) | 0.9681 ± 0.0413 (0.9733) | 0.9954 ± 0.0066 (1.0000) |  |
| Xception | 0.9984 ± 0.0056 (0.9999) | 0.9885 ± 0.0172 (0.9931) | 0.9657 ± 0.0465 (0.9733) | 0.9938 ± 0.0072 (1.0000) |  |
| Inception-ResNet V2 | 0.9926 ± 0.0074 (0.9957) | 0.9802 ± 0.0089 (0.9826) | 0.9534 ± 0.0362 (0.9600) | 0.9863 ± 0.0068 (0.9906) |  |

* The results are expressed as the mean ± standard deviations (the optimal value).

AUC, area under the curve; DCs, dendritic cells
